# Cost-effectiveness of immune checkpoint inhibitors as a first-line therapy for advanced hepatocellular carcinoma: a systematic review

**DOI:** 10.1186/s13561-024-00526-2

**Published:** 2024-07-05

**Authors:** Hongyu Gong, Siew Chin Ong, Fan Li, Yan Shen, Zhiying Weng, Keying Zhao, Zhengyou Jiang, Meng Wang

**Affiliations:** 1 Incubation Center for Scientific and Technological Achievements, Kunming Medical University, Chunrong west road 1168, Kunming City, China; 2 School of Pharmaceutical Sciences, Universiti Sains Malaysia, 11800 USM Penang, Penang City, Malaysia; 3 Gastroenterology Oncology, Yunnan Cancer Hospital, Kunzhou Road 519, Kunming City, China; 4 School of Pharmaceutical Science &Yunnan Key Laboratory of Pharmacology for Natural Products, Kunming Medical University, Chunrong west road 1168, Kunming City, China; 5 School of Public Health, Kunming Medical University, Chunrong West Road 1168, Kunming City, China; 6 School of Management, Universiti Sains Malaysia, 11800 USM Penang, Penang City, Malaysia; 7 Physical Examination Center, Kunming Center for Disease Prevention and Control, Xichang Road 126, Kunming City, China; 8 Yunnan Drug Policy Research Center, Kunming Medical University, Kunming, China

**Keywords:** Cost-effectiveness, Health technology assessment, Immune checkpoint inhibitors, Advanced hepatocellular carcinoma, Systematic review

## Abstract

Since 2017, immune checkpoint inhibitors (ICIs) have been available for the treatment of advanced hepatocellular carcinoma (HCC) or unresectable HCC, but their adoption into national medical insurance programs is still limited. Cost-effectiveness evidence can help to inform treatment decisions. This systematic review aimed to provide a critical summary of economic evaluations of ICIs as a treatment for advanced HCC and identify key drivers (PROSPERO 2023: CRD42023417391). The databases used included Scopus, Web of Science, PubMed, Embase, and Cochrane Central. Economic evaluations of ICIs for the treatment of advanced HCC were included. Studies were screened by two people. Of the 898 records identified, 17 articles were included. The current evidence showed that ICIs, including atezolizumab plus bevacizumab, sintilimab plus bevacizumab/bevacizumab biosimilar, nivolumab, camrelizumab plus rivoceranib, pembrolizumab plus lenvatinib, tislelizumab, durvalumab, and cabozantinib plus atezolizumab, are probably not cost-effective in comparison with tyrosine kinase inhibitors or other ICIs. The most influential parameters were price of anticancer drugs, hazard ratios for progression-free survival and overall survival, and utility for health statest. Our review demonstrated that ICIs were not a cost-effective intervention in advanced HCC. Although ICIs can significantly enhance the survival of patients with advanced HCC, decision-makers should consider the findings of economic evaluations and affordability before adoption of new therapies.

**Supplementary Information:**

The online version contains supplementary material available at 10.1186/s13561-024-00526-2.

## Introduction

Liver cancer is the third most common cause of cancer death. Liver cancer caused 830,180 deaths globally in 2020, with the most common form of liver cancer being hepatocellular carcinoma (HCC) [1]. In East Asia, North Africa, Micronesia, Southeast Asia, and Melanesia, the age-standardized incidence and mortality rates of HCC are much higher than the world average (i.e., age-standardized incidence and the mortality rate is 9.5 per 100,000 and 8.7 per 100,000 of the population, respectively) [1]. There are many treatments for early stage HCC, including surgery (liver resection or liver transplantation), thermal ablation, intra-arterial therapies, and stereotactic body radiotherapy, but patients are often diagnosed with HCC in an advanced stage or without the opportunity for localised treatment [1–6].

While various treatment modalities exist for early-stage HCC, the management of advanced HCC predominantly revolves around systemic therapy [7, 8]. Sorafenib, a tyrosine kinase inhibitor (TKI), emerged as a pivotal first-line treatment following notable clinical trials [9]. Subsequent studies have explored alternative TKIs [10, 11] and, more recently, immune checkpoint inhibitors (ICIs), which have revolutionized HCC treatment paradigms.

ICIs, such as PD-1/PD-L1 inhibitors, have demonstrated remarkable efficacy in improving overall survival (OS) rates, as evidenced by numerous clinical trials [8, 12, 13]. Notable among these are studies on atezolizumab plus bevacizumab [14], cabozantinib plus atezolizumab [15], sintilimab plus a bevacizumab biosimilar (IBI305) [16], tislelizumab [17], camrelizumab plus rivoceranib [18], pembrolizumab plus lenvatinib [19, 20], nivolumab [21], and durvalumab plus tremelimumab [22]. However, despite their clinical promise, the widespread adoption of ICIs is impeded by significant financial implications for both individuals and healthcare systems.

The economic burden is exacerbated by the need for advanced and costly treatments, such as immunotherapies, which are frequently part of the standard of care for advanced HCC. Given the limited availability of healthcare resources, cost-effectiveness analysis can assist healthcare institutions and governmental agencies in better resource allocation [23]. By evaluating the cost-effectiveness of different medications, it can determine which regimens can maximize health outcomes within a given budget. The pharmacoeconomic evidence on advanced HCC has been emerging in several reviews which primarily focused on TKIs [24, 25]. Hence, aggregating the latest pertinent pharmacoeconomic studies would be valuable to achieve a more thorough understanding of the currently approved ICIs for advanced HCC.

Although ICIs have health benefits as a first-line treatment for advanced HCC, their use is limited because of the financial burden to individuals and governments. This study aims to delve into the cost-effectiveness of ICIs as first-line therapy for advanced HCC. The objective of this present review is to outline the essential features and outcomes of cost-effectiveness evidence, aiming to furnish decision-makers with pertinent information.

## Methods

This systematic review was performed according to the Preferred Reporting Items for Systematic Reviews and Meta-analyses (PRISMA) statement (Supplementary Table 1) [26]. The systematic review was registered on the international prospective register of systematic reviews (PROSPERO) database (CRD42023417391).

### Data sources and search strategy

We searched Scopus, Web of Science, PubMed, Embase, and Cochrane Central databases. Additionally, a comprehensive snowball manual search was conducted, encompassing the perusal of citations within eligible studies and pertinent reviews. The search methodology employed both free texts and subject headings to explore about HCC, drug or therapy, and pharmacoeconomic assessments. EndNote 20 software facilitated the systematic recording and organization of retrieved articles, streamlining the deduplication process and facilitating efficient screening. The search spanned from January 1, 2010, to April 1, 2024, with inclusion criteria limited to studies published in the English language. Detailed search strategies for each database can be found in Supplementary Table 2.

### Eligibility criteria

In accordance with the defined scope and objectives of our study, eligibility criteria were pre-established as delineated in Supplementary Table 3. Our focus primarily centered on the inclusion of original comprehensive pharmacoeconomic evaluations concerning ICIs as first-line treatment of advanced HCC.

### Selection

According to the eligibility criteria, two reviewers (Hongyu Gong and Zhengyou Jiang) independently screened titles and abstracts to identify potentially relevant studies. Subsequently, the full texts of articles meeting the eligibility criteria were examined by both reviewers to determine their final inclusion. Throughout both phases of screening, reasons for exclusion were documented. Any discrepancies in the inclusion of studies were resolved through thorough discussion and mutual consensus between the two reviewers.

### Data extraction

All outcome variables reported in the included studies were extracted into a pre-specified data extraction Excel form. The selected articles were examined to extract essential information, including participants, interventions, comparators, outcomes, study types, and funding sources. First, one reviewer conducted the data extraction, which was subsequently reviewed and verified by another reviewer to mitigate any potential omissions or errors.

### Synthesis of results

Given the heterogeneity of the available evidence, a qualitative, descriptive approach was employed to evaluate the aggregated findings from economic studies regarding ICIs as first-line treatment for advanced HCC. The economic evaluation methodologies utilized in each study were classified into four distinct approaches. These include cost-minimization analysis (CMA), cost-effectiveness analysis (CEA), cost-benefit analysis (CBA), and cost-utility analysis (CUA) [27]. The costs analyzed in this systematic review were converted into US dollars ($) using the CCEMG-EPPI-Centre Cost Converter v.1.6, available online at https://eppi.ioe.ac.uk/costconversion/.

A narrative synthesis of the data was conducted, summarizing all findings through tables and figures. Primary outcomes encompassed the incremental cost-effectiveness ratio (ICER), and key drivers of economic evaluations. Secondary outcomes examined the economic evaluation type, scenario analysis, and the particularities of each study.

### Quality assessment

The Consolidated Health Economic Evaluation Reporting Standards (CHEERS) Checklist [28] was employed to assess the quality of the included studies on the 28 items (Supplementary Table 4). The full-text articles were evaluated against the 28 items with ‘yes’ if they reported the relevant information and ‘no’, if not. The percentages of the studies reporting the items were calculated to obtain a general view of the completeness and quality of the studies.

## Results

### Selection of the included studies

**Fig. 1:**
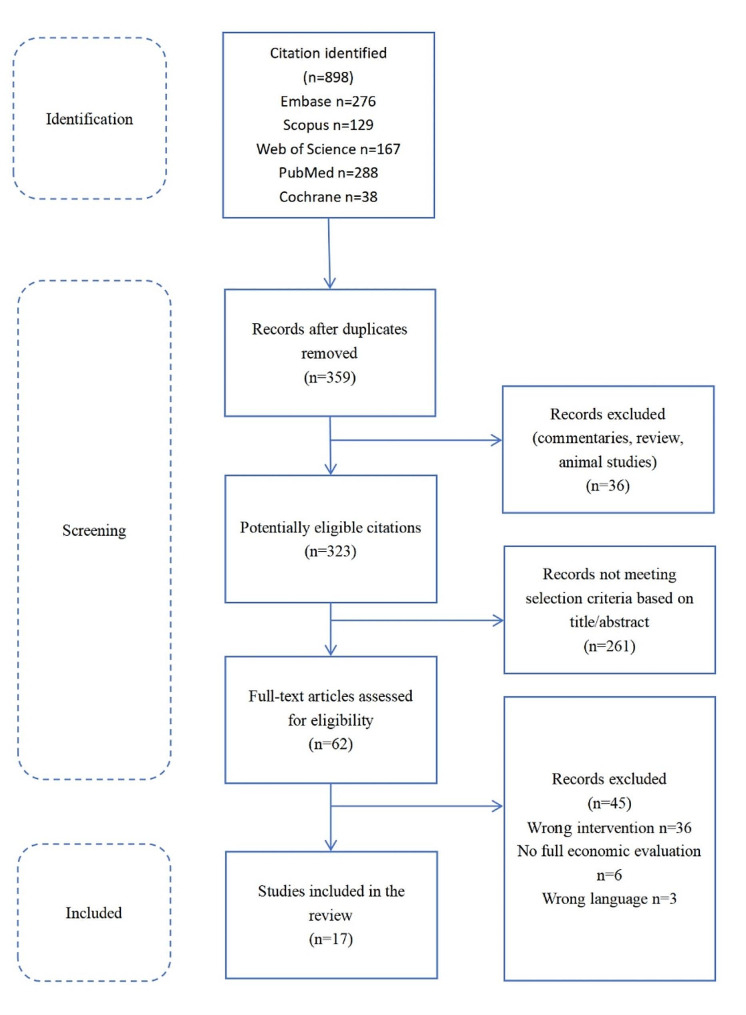
Flow diagram of the selection process

A total of 898 records were identified through database searching, and 359 records remained after duplicates were removed. Of these, 261 records were deemed ineligible based on their title and abstract. Of the 62 records that qualified for a full-text review, 45 full-text articles were excluded owing to wrong intervention, not full economic evaluation and non-English language. Finally, a total of 17 studies were selected for this systematic review (Fig. 1) [29–41].

### Quality assessment of the included studies

The quality assessment of the 17 full-text articles included in the review was conducted using the CHEERS Checklist. Percentages reflecting the reporting of the 28 checklist items computed and presented in Supplementary Table 4. Each of the included studies provided a comprehensive depiction of their study context and settings, outlined the objectives of the economic evaluation, described the interventions or strategies under investigation, outlined baseline characteristics, selected a particular model structure and offered a detailed description of the model. Furthermore, all full-text articles detailed the measurement and estimation of health outcomes, resources, and costs.

However, only 41.0% of the studies indicated that a health economic analysis plan was developed, and 82.0% of the studies stated the time horizon for the study. In the results section, a summary of the major study parameters and main findings from the review was provided. Additionally, the effect of uncertainty was addressed and discussed in all studies. Limitations and generalizability of the full-text studies were explicitly clarified. Notably, 29% of the studies explicitly engaged patients or other stakeholders affected by the study—a focus that is now emphasized in the latest version of the CHEERS checklist. All full-text studies both disclosed their funding sources and declared conflicts of interest. Further details of the quality assessment are provided in Supplementary Table 5.

### Baseline characteristics of the included studies

The general characteristics of the included studies are presented in Table 1 (the details of the Baseline Characteristics of the studies have been provided in Supplementary Table 6). All studies included collation of a cost-effectiveness analysis with quality-adjusted life-years (QALYs) as the health outcome [29–45].

**Table 1:** Overview of published economic evaluations of immune checkpoint inhibitors as a first-line therapy for advanced hepatocellular carcinoma

First author, year (country)	Country	Interventions	Comparator	Perspective	Funding source	Model	Discount rate (%)	Efficacy source	Trial name	Trial setting
Zhao, M, 2022 (China)	China	Sintilimab - Bevacizumab, and Atezolizumab - Bevacizumab	Sorafenib	Healthcare system	The government (China)	Partitioned survival model	5%	No	IMbrave150, REFLECT, ORIENT-32, and ZGDH3	RCTs
Zhou, T, 2022 (China)	China	Sintilimab - IBI305	Lenvatinib	Healthcare system	Manufacturing company and the government (China)	Partitioned survival model	5%	No	ORIENT-32 and REFLECT	RCTs
Su, D, 2021 (US)	The US	Atezolizumab - Bevacizumab	Sorafenib	Payer perspective	The government (China)	Partitioned survival model	3%	No	IMbrave150	RCTs
Zhang, X, 2021 (China)	The US	Atezolizumab - Bevacizumab	Sorafenib	Payer perspective	The government (China)	Partitioned survival model	3%	No	IMbrave150	RCTs
Gaugain, L., 2023 (France)	France	Atezolizumab - Bevacizumab	Sorafenib	Healthcare system	Manufacturing company	Partitioned survival model	2.50%	Yes	IMbrave150	RCTs and the real-life long-term data.
Chiang, C. L, 2021 (China, Hongkong)	The US	Atezolizumab - Bevacizumab	Sorafenib	Payer perspective	None	Markov model	3%	No	IMbrave150	RCTs
Sun, K. X, 2022 (China)	China and the US	Sintilimab - Bevacizumab, and Atezolizumab - Bevacizumab, Nivolumab, Donafenib	Donafenib	Payer perspective	The government (China)	Markov model	In China: 5% In the United States: 3%	No	NR	RCTs
Li, L, 2022 (China)	China	Sintilimab - IBI305, and Atezolizumab - Bevacizumab	Sorafenib	Healthcare system	None	Partitioned survival model	5%	No	ORIENT-32 and IMbrave 150	RCTs
Li, Y, 2022 (China)	The US	Nivolumab	Sorafenib	Third-party payer perspective	The government (China)	Partitioned survival model	3%	No	CheckMate 459	RCTs
Li, Y, 2022 (China)	The US	Atezolizumab - Bevacizumab	Nivolumab	Societal perspective	The government (China)	Partitioned survival model	3%	No	IMbrave150 and CheckMate 459	RCTs
Wen, F, 2021 (China)	China and the US	Atezolizumab - Bevacizumab	Sorafenib	Payer perspective	The government (China)	Markov model	3%	No	ORIENT-32	RCTs
Zhou, T, 2022 (China)	China	Sintilimab - Bevacizumab	Sorafenib	Healthcare system	Manufacturing company	Partitioned survival model	5%	No	ORIENT-32	RCTs
Liu, K. 2023 (China)	China	Sintilimab - IBI305, Atezolizumab - Bevacizumab, Camrelizumab - Rivoceranib, Pembrolizumab-Lenvatinib, Nivolumab, Tislelizumab, Durvalumab, Cabozantinib - Atezolizumab	Sorafenib	Payer perspective	The university fund (China)	Markov model	3%	No	ORIENT-32; IMbrave150; CheckMate 459; RATIONALE-301; SHR-1210-III-310; and LEAP-002	RCTs
Zheng, Z 2024 (China)	China	Tslelizumab	Sorafenib	Payer perspective	None	Partitioned survival model	5%	No	RATIONALE-301	RCTs
Sriphoosanaphan, 2024, (Thailand)	Thailand	Atezolizumab - Bevacizumab	Best supportive care	Societal perspective	Manufacturing company and the university fund (Thailand)	Markov model	3%	No	IMbrave150	RCTs
Lang W, 2024, (China)	China	Camrelizumab - rivoceranib	Sorafenib	Healthcare system	None	Markov model	5%	No	CARES-310	RCTs
Gong H, 2023, (China)	China	Atezolizumab - Bevacizumab, Sintilimab - Bevacizumab	Sorafenib	Chinese patient rspective	The government (China)	Partitioned survival model	5%	No	IMbrave150, ORIENT-32, REFLECT,	RCTs

RCTs: randomized controlled trials; IBI305: bevacizumab biosimilar; the US: the United States

The majority of studies were carried out in the United States (US) and China settings [29, 31–42, 44, 45], with an additional 2 studies conducted in France [30], or Thailand [43]. 2 studies examined the cost-effectiveness of ICIs as first-line treatment for advanced HCC in both the US and China [36, 37]. 7 studies examined the costs and QALYs of atezolizumab plus bevacizumab as an intervention for first-line treatment of advanced HCC relative to sorafenib or best supportive care [29, 30, 32, 35, 37, 38, 43]. The cost-effectiveness of sintilimab plus bevacizumab/bevacizumab biosimilar was evaluated in 2 studies [40, 41]. The cost-effectiveness analysis compared nivolumab [33], tslelizumab [42] and camrelizumab plus rivoceranib [44] with sorafenib, respectively. 5 studies conducted cost-effectiveness analysis of various ICIs, including atezolizumab plus bevacizumab, sintilimab plus bevacizumab/bevacizumab biosimilar, camrelizumab plus rivoceranib, pembrolizumab plus lenvatinib, nivolumab, tislelizumab, durvalumab, or cabozantinib plus atezolizumab [31, 34, 36, 39, 45].

8 studies were from the payer perspective, whereas the third-party payer perspective was used in one study [29, 33–38, 42]. The costs for the perspective were direct costs, including the costs of drugs, costs attributed to the patient’s health state, costs for the management of adverse drug reactions, and costs for end-of-life care. 6 studies were conducted from a health system perspective [30, 31, 39–41, 44], and only the direct medical cost of the patient was considered. 2 studies used the societal perspective [32, 43]. In addition to formal healthcare sector costs, the societal perspective model incorporated informal healthcare costs, such as patient time and/or salary, transportation, and caregiver costs. A study used the patient’s perspective, where the cost was calculated based on medical insurance reimbursement [45].

All of the studies, through their full-text research articles, either disclosed their funding sources, conflicts of interest, or both. 4 of these 13 studies were not funded [29, 31, 42, 44], whereas the rest received funding from several sources. The majority of studies were funded by the Chinese government or university funds [32–39, 45], with four involving relevant pharmaceutical companies (such as Roche, Innovent Biologics, etc.) in different capacities [30, 40, 41, 43].

Different decision-analytic methodologies were identified in the modeling-based research. In terms of model structure, 11 studies involved construction of a partitioned survival model based on three mutually exclusive health states: progression-free survival (PFS), progressive disease, and death [30–33, 35, 38–42, 45]. 6 studies of three-state Markov models were developed based on the disease progression of advanced HCC: PFS, progressive disease, and death [29, 34, 36, 37, 43, 44]. The hypothetical target population for these analyse was assumed to be consistent with the patient characteristics of randomized controlled trials (RCTs). Discount rates ranged from 2.5 to 5%.

Local data were used in cases where they were available. In all studies, clinical data for PFS and OS were obtained from phase III clinical trials, except for one study from France that integrated survival data using additional ATHENOR data [46]. ATHENOR is a database related to population characteristics, management, and survival of French individuals with HCC [46]. 10 phase III clinical trials were direct head-to-head comparisons based on survival data [29, 30, 33, 35, 37, 38, 40, 42–44]. Owing to the inclusion of multiple drug cost-effectiveness comparisons, 7 studies used an indirect comparison method to calculate the hazard ratios (HRs) of PFS and OS for the regimens [31, 32, 34, 36, 39, 41, 45]. For the aspect of utility, country-specific utility data for cost-effectiveness were used in the French study [30]. For the Chinese and US studies, the utilities of different advanced HCC first-line treatments were mainly obtained from the literature [31, 34, 36, 37, 39–41]. In all cost studies, the prices were derived from individual national data.

### Study results

#### Outcomes of cost-effectiveness analysis

The modelling-based studies adopted various time horizons, among which 4 stretched the evaluation to a lifetime [35, 40, 41, 45], 10 studies projected the outcomes in a period of 5 to 10 years [29, 32, 33, 36–39, 42–44], 2 studies selected a time horizon 15 years [30, 31] (Table 2).

**Table 2:** Results of base-case analysis of advanced hepatocellular carcinoma

First author, year (country)	Costing year	Time horizon (years)	Cost (USD, $)	QALY	Life-years	ICER ($/QALY)	WTP (USD, $)	Cost-effectiveness
Zhao, M, 2022 (China)	2021	10					3* GDP: 33,521 per QALY gained	
Sorafenib			16,614.86	0.91	1.38	/		/
Sintilimab - Bevacizumab			43,195.21	1.42	2.33	51,877.36		Not cost-effective
Atezolizumab - Bevacizumab			129,281.72	1.77	2.84	130,508.44		Not cost-effective
Zhou, T, 2022 (China)	2021	Lifetime					1* GDP: 12,516 per QALY gained ; 3* GDP: 37,547 per QALY gained	
Lenvatinib			21,037	0.938	1.32	/		/
Sintilimab - IBI305			33,102	1.431	2.04	24,462		1* GDP: Not cost-effective; 3* GDP: Cost-effective
Su, D, 2021 (US)	2019	Lifetime					150,000 per QALY gained	
Sorafenib			202,973	1.021	1.736	/		/
Atezolizumab - Bevacizumab			292,780	1.551	3.033	169,223		Not cost-effective
Zhang, X, 2021 (China)	2020	6					100,000 per QALY gained	
Sorafenib			156,984	0.928	1.218	/		/
Atezolizumab - Bevacizumab			313,193	1.412	1.840	322,500		Not cost-effective
Gaugain, L., 2023 (France)	2017	15					133,775 per QALY gained	
Sorafenib			30,189	1.35	1.57	/		/
Atezolizumab - Bevacizumab			129,363	1.95	2.26	163,651		Not cost-effective
Chiang, C. L, 2021 (China, Hongkong)	2020	5					100,000 per QALY gained; 150,000 per QALY gained	
Sorafenib			634,668	0.987	1.51	/		/
Atezolizumab - Bevacizumab			713,742	1.426	2.02	179,729		Not cost-effective
Sun, K. X, 2022 (China)	2021	10						
China							1* GDP: 11,101.70 per QALY gained	
Donafenib			5,604.64	8.77	/	/		/
Atezolizumab - Bevacizumab			44,744.51	9.23	/	85,607.88		Not cost-effective
Sintilimab - Bevacizumab			20,697.68	10.02	/	12,109.27		Not cost-effective
The United States							69,375 per QALY gained	
Nivolumab			119,603.30	9.86	/	/		/
Atezolizumab - Bevacizumab			299,542	13.61	/	47,896.93		Cost-effective
Li, L, 2022 (China)	2020	15					3* GDP: 33,500 per QALY gained	
Sorafenib			18,567.66	1.11	1.59	/		/
Sintilimab - IBI305			43,109.99	1.73	2.47	39,766.86		Not cost-effective
Atezolizumab - Bevacizumab			79,965.01	1.71	2.45	103,037.66		Not cost-effective
Li, Y, 2022 (China)	2022	10					150,000 per QALY gained	
Sorafenib			320,536	1.27	1.95	/		/
Nivolumab			390,298	1.59	2.45	220,864		Not cost-effective
Li, Y, 2022 (China)	2022	10					150,000 per QALY gained	
Nivolumab			390,220	1.59	2.45	/		/
Atezolizumab - Bevacizumab			468,500	2.27	3.58	113,892		Cost-effective
Wen, F, 2021 (China)	2020	10						
China							3* GDP: 28,527 per QALY gained	
Sorafenib			18,833.34	0.87	1.22	/		/
Atezolizumab - Bevacizumab			95,972.83	1.40	1.96	145,546.21		Not cost-effective
The United States							150,000 per QALY gained	
Sorafenib			194,248.14	0.87	1.22	/		/
Atezolizumab - Bevacizumab			283,304.15	1.40	1.96	168,030.21		Not cost-effective
Zhou, T, 2022 (China)	2021	Lifetime					3* GDP: 33,592 per QALY gained	
Sorafenib			23,294	0.928	/	/		/
Sintilimab - Bevacizumab			33,766	1.428	/	20,968		Cost-effective
Liu, K. 2023 (China)	2022	15					3* GDP: 37,653 per QALY gained	
Sorafenib			28,746	1.289	1.837	/		/
Cabozantinib - Atezolizumab			56,396	1.410	1.994	228,512		Not cost-effective
Durvalumab			33,972	1.498	2.128	25,005		Cost-effective
Tislelizumab			26,808	1.509	2.149	− 8,809		Cost-effective
Nivolumab			32,703	1.515	2.148	17,509		Cost-effective
Pembrolizumab - Lenvatinib			44,731	1.594	2.260	52,410		Not cost-effective
Camrelizumab - Rivoceranib			40,307	1.795	2.603	22,848		Cost-effective
Atezolizumab - Bevacizumab			73,457	1.870	2.646	76,955		Not cost-effective
Sintilimab - IBI305			56,259	2.076	2.950	34,959		Cost-effective
**Zheng, Z 2024 (China)**	2022	10					3* GDP: 37304.34 per QALY gained	
Sorafenib			14306.87	1.06	/	/		/
Tislelizumab			16181.24	1.24	/	10,413.17		Cost-effective
Sriphoosanaphan, 2024, (Thailand)	2024	5					4,678 per QALY gained	
Best supportive care			3,312	0.4051	/	/		/
Atezolizumab - Bevacizumab			48,669	0.8401	/	54,589		Not cost-effective
**Lang W, 2024, (China)**	2024	10					3* GDP: 35,864.61 per QALY gained	
Sorafenib			16,800.92	1.52	/	/		/
Camrelizumab plus Rivoceranib			30,485.76	1.93	/	33,619.98		Cost-effective
**Gong H, 2023, (China)**	2023	Lifetime					3* GDP: 36,600 per QALY gained	
Sorafenib			16,109.80	1.30	/	/		/
Sintilimab-Bevacizumab			39,406.40	1.61	/	75,150.32		Not cost-effective
Atezolizumab-Bevacizumab			141,836.73	2.17	/	144,513.71		Not cost-effective

QALY: quality-adjusted life-year; ICER: incremental cost effectiveness ratio; IBI305: bevacizumab biosimilar; GDP: per capita gross domestic product; USD: United States Dollar;

In 10 studies, despite the better health effects of ICIs, TKIs were still the most cost-effective first-line drugs in advanced HCC because of the low prices [29–31, 33, 35, 37–39, 43, 45] (Table 2). In 5 studies, ICIs were considered to be cost-effective [34, 40–42, 44]. One study found that the use of ICIs in China was not cost-effective, while the opposite was found in the US [36].

In 8 studies, a cost-effectiveness threshold of 1–3 times per capita gross domestic product (GDP) was stated based on the cost per QALY gained, according to Chinese guidelines for pharmacoeconomic evaluation [31, 34, 39–42, 44, 45, 47]. In 5 studies, individual country-specific thresholds of $150,000 per QALY gained in the US [32, 33, 35], $133,775 per QALY gained in France [30], and $4,678 per QALY gained in Thailand [43] were used to perform the analysis. 2 cost-effectiveness studies were conducted using $100,000 per QALY and $150,000 per QALY gained in the US [29, 38]. In one study, the threshold was set at 1 time per capita GDP in China and $69,375 in the US [36]. In another study, the threshold was set at 3 times per capita GDP in China and $150,000 in the US [37].

Economic evaluation results were shown in Table 2. The cost-effectiveness thresholds were 1–3 times per capita GDP in the Chinese studies [31, 34, 36, 37, 39–42, 44, 45]. When the threshold was $11101.70 per QALY gained (1 time the per capita GDP) in China, the ICI was not cost-effective in a 2021 study [36]. A similar outcome was observed in the study of a ICI, where the regimen was not considered cost-effective when the threshold was set at 1 time the per capita GDP in 2021 ($12,516), but became cost-effective at 3 times the per capita GDP ($37,547) [41]. In 4 studies, when the willing to pay (WTP) thresholds was 3 times the per capita GDP ($28,527 per QALY gained in 2019 [37], $33,521 per QALY gained in 2020 [39], $33,500 per QALY gained in 2020 [31] and $36,600 per QALY gained in 2023 [45], respectively), ICIs were not considered as an economical option. However, there were 4 other studies that indicated ICIs could be considered cost-effective choices at 3 times the per capita GDP with WTP thresholds in China being set at $33,592 per QALY in 2021 [40], $37,653 per QALY gained in 2022 [34], $37,304.34 per QALY gained in 2022 [42], and $35,864.61 per QALY gained in 2024 [44]. In the US studies, the cost-effectiveness thresholds were $69,375 per QALY gained, $100,000 per QALY gained, or $150,000 per QALY gained [29, 32, 33, 35–38]. In one US study, ICIs were not the favorite choice when the threshold was $69,375 per QALY gained [36]. No study considered ICIs as a cost-effective option when the threshold was $100,000 per QALY gained [29, 38]. When the threshold was $150,000 per QALY gained, four studies considered that ICIs were not cost-effective [29, 33, 35, 38], but one study considered ICIs as cost-effective [32]. The threshold of $133,775 per QALY gained was studied in France [30]. In base case analysis, ICI vs. TKI led to an incremental cost-effectiveness ratio of $166,221/QALY, which is over the threshold; however, ICI was considered as a cost-effective strategy after adjusting for survival. In 2024, with a Thai threshold of $4,678 per QALY gained, the ICER of the ICI continued to exceed the threshold as well [43].

#### Sensitivity and uncertainty analysis

All studies included both one-way sensitivity and probabilistic sensitivity analyses (Table 3 and Supplementary Table 7). Cost of drug was consistently identified as a significant driver across multiple studies, indicating its substantial impact on the cost-effectiveness of treatments [31–37, 39–45, 48]. The HRs for OS and PFS emerged as crucial drivers affecting the economic outcomes, underscoring their importance in determining treatment efficacy and cost-effectiveness [29, 31, 32, 34, 35, 38, 39, 41, 43]. Other important drivers included the utility of different health states (e.g., PFS, and progression disease) [31, 33, 36, 37, 40, 44, 45], body weight [29, 32, 35, 38], cost of subsequent treatment [40–42], discount rates for benefits and costs [30, 36], and percentage of patients receiving subsequent treatment [30, 44]. Table 4 shows the influential parameters reported in each study of economic evaluations of first-line treatment of advanced HCC.

**Table 3:** Sensitivity analyses performed in economic evaluations of the first-line treatment of advanced hepatocellular carcinoma

First author, year (country)	Sensitivity analyses	Important drivers			Type of scenario analysis	Outcomes of scenario analysis
		1st	2nd	3rd		
Zhao, M, 2022 (China)	1-way SA, PSA, scenario analysis	HR for OS	HR for PFS	Cost of drug	Patients receive active treatment until death.	Not cost-effective
Zhou, T, 2022 (China)	1-way SA, PSA, scenario analysis	HR for OS	Cost of drug	Cost of subsequent treatment	A reduced dose of bevacizumab and its biosimilar is administered due to treatment intolerance.	1* GDP: Cost-effective; 3* GDP: Cost-effective
Su, D, 2021 (US)	1-way SA, PSA	HR for OS	Cost of drug	Body weight	/	/
Zhang, X, 2021 (China)	1-way SA, PSA	HR for OS	Body weight	HR for PFS	/	/
Gaugain, L., 2023 (France)	1-way SA, PSA, scenario analysis	Discount rate for benefits	Discount rate for costs	Percentage of patients receiving subsequent treatment	Integration of survival using additional ATHENOR data.	Cost-effective
Chiang, C. L, 2021 (China, Hongkong)	1-way SA, PSA, scenario analysis	HR for OS	Body weight	Cost of drug	In the pessimistic scenario, the survival estimates of the US population with advanced HCC from the SEER database. In the optimistic scenario, all patients “alive” at 17 months were “cured,” with their risk of death equal to their age-adjusted background mortality rate.	Pessimistic scenario: Not cost-effective; Optimistic scenario: Cost-effective
Sun, K. X, 2022 (China)	1-way SA, PSA, scenario analysis	Discount rate	Cost of drug	Utility of PFS	Drug donation programs in Chinese low-income patients.	Not cost-effective
Li, L, 2022 (China)	1-way SA, PSA, scenario analysis	HR for OS	Utility of PD	Cost of drug	Patient assistance program	Sintilimab - IBI305: Cost-effective; Atezolizumab - Bevacizumab: Not cost-effective
Li, Y, 2022 (China)	1-way SA, PSA	Cost of drug	Utility of PD	Utility of PFS	/	/
Li, Y, 2022 (China)	1-way SA, PSA	HR for PFS	Cost of drug	Body weight	/	/
Wen, F, 2021 (China)	1-way SA, PSA	Cost of drug	Utility of PD	Utility of PFS	The price of atezolizumab was 30% of the primary price.	Cost-effective
Zhou, T, 2022 (China)	1-way SA, PSA, scenario analysis	Cost of subsequent treatment	Cost of drug	Utility of PFS	A reduced dose of bevacizumab and its biosimilar is administered due to treatment intolerance.	Cost-effective
Liu, K. 2023 (China)	1-way SA, PSA	HR for OS	Cost of drug	HR for PFS	/	/
Zheng, Z, 2024 (China)	1-way SA, PSA	Cost of subsequent treatment	Cost of drug	Utility of PD	/	/
Sriphoosanaphan, 2024, (Thailand)	1-way SA, PSA, scenario analysis	HR for OS	HR for PFS	Cost of drug	The WTP threshold raises to $60,819 per QALY gained.	Cost-effective
Lang W, 2024, (China)	1-way SA, PSA, scenario analysis	Cost of drug	Percentage of subsequent treatment	Utility of PFS	Patients with albumin-bilirubin grade 1, and grade 2	ALBI grade 1: The ICER was close to the WTP threshold; ALBI grade 2: Cost-effective
Gong H, 2023, (China)	1-way SA, PSA	Drug reimbursement ratio	Cost of drug	Utility of PFS	/	/

PSA: probabilistic sensitivity analysis; SA: Sensitivity analysis; OS: Overall survival; PFS: progression-free survival; HR: Hazard rate; PD: Progressive disease; GDP: per capita gross domestic product; SEER: Surveillance, Epidemiology, and End Results;

**Table 4:** Influential parameters reported in each study of economic evaluation of first-line treatment of advanced hepatocellular carcinoma

First author, year (country)	Antitumor drug price	Drug dosage	OS HR	PFS HR	Parameters for survival model	utility for PFS	utility for OS	Cost for subsequent treatment	Discount rate	Proportion of receiving subsequent therapy	Body Weight	Cost of ADR	Probability of ADR grade 3 − 5	Disutility of ADR	Follow-up cost	Proportion of insurance
Zhao, M, 2022 (China)	Y	Y	Y	Y	Y	Y	Y			Y						
Zhou, T, 2022 (China)	Y		Y	Y				Y	Y							
Su, D, 2021 (US)	Y		Y	Y		Y	Y	Y		Y	Y	Y	Y		Y	
Zhang, X, 2021 (China)	Y		Y	Y		Y	Y			Y	Y					
Gaugain, L., 2023 (France)	Y					Y	Y	Y	Y	Y			Y		Y	
Chiang, C. L, 2021 (China, Hongkong)	Y		Y	Y		Y	Y	Y			Y					
Sun, K. X, 2022 (China)	Y					Y	Y	Y	Y			Y			Y	
Li, L, 2022 (China)	Y		Y	Y		Y	Y	Y	Y	Y	Y					
Li, Y, 2022 (China)	Y		Y	Y	Y	Y	Y	Y				Y		Y	Y	
Li, Y, 2022 (China)	Y		Y	Y		Y	Y	Y			Y	Y		Y	Y	
Wen, F, 2021 (China)	Y				Y	Y	Y					Y			Y	
Zhou, T, 2022 (China)	Y					Y	Y	Y	Y			Y	Y	Y	Y	
Liu, K. 2023 (China)	Y		Y	Y		Y	Y					Y	Y	Y	Y	
Zheng, Z, 2024 (China)	Y					Y	Y	Y	Y			Y		Y	Y	
Sriphoosanaphan, 2024, (Thailand)	Y		Y	Y		Y	Y	Y	Y							
Lang W, 2024, (China)	Y					Y	Y	Y	Y	Y		Y		Y	Y	
Gong H, 2023, (China)	Y					Y	Y	Y	Y			Y			Y	Y

Y: Yes

Scenario analyses were carried out in 11 studies [29–31, 35–37, 39–41, 43, 44]. Two scenario analyses turned out to demonstrate the cost-effectiveness of combining sintilimab with a reduced dosage of bevacizumab, which was set at 7.5 mg/kg instead of the standard 15 mg/kg due to treatment intolerance [40, 41]. Gaugain et al. concluded that the inclusion of primary conditional survival based on ATHENOR after 20 months resulted in the ICI being deemed cost-effective [30]. Furthermore, The ICI will be considered as a cost-effective strategy when the scenario assumed that all patients who were “alive” at 17 months were considered “cured,” with their risk of death equal to their age-adjusted background mortality rate, which corresponds to a 3-year survival rate of 60.7% [29]. At the same time, providing a patient assistance program [31], setting the price of atezolizumab at 30% of the primary price [37], and raising the WTP threshold to $60,819 per QALY gained [43] can all potentially change some base-case analyses from being not cost-effective to cost-effective in the scenario analyses. However, certain scenario analyses, such as “Patients received active treatment until death [39]” and “Drug donation programs in Chinese low-income patients [36],” still indicate that ICI treatment is not cost-effective.

## Discussion

Quality assessment using the latest CHEERS checklist reveals that all aspects of reporting are addressed in the majority of studies, with some variations in adherence to specific guidelines. Notably, the title, abstract, introduction, comparators, perspective, selection of outcomes, measurement and valuation of outcomes, and study parameters were consistently reported in 100% of the studies. However, there are lower adherence rates for certain aspects, such as the development of a health economic analysis plan (41%), characterizing heterogeneity (52%), and describing the approach to engagement with patients or stakeholders in the study design (29%). Currently, worldwide efforts toward Patient and Public Involvement and Engagement (PPIE), broader community engagement, and stakeholder involvement in health economic evaluation are still in the early stages, with the goal of bolstering the relevance, acceptability, and appropriateness of research, thereby enhancing its overall quality [28, 49]. These findings suggest overall robust reporting practices but also indicate areas for improvement, particularly in addressing heterogeneity and engaging stakeholders in the research process.

The current review comprehensively consolidated the pharmacoeconomic evidence relevant to first-line regimens of ICIs for advanced HCC. To the best of our knowledge, no comprehensive review of the cost-effectiveness of ICIs for the treatment of advanced HCC has been published that evaluates quality assessment and methodological approaches. We included 17 complete ICI economic evaluations in this systematic review. A study showed that nivolumab was not cost-effective in the US [33]. 2 studies have demonstrated that atezolizumab plus bevacizumab is likely considered cost-effective as a first-line treatment for advanced HCC in the US [32, 36]. However, 4 studies indicate that the combination of atezolizumab and bevacizumab is not cost-effective [29, 35, 37, 38]. At the same time, atezolizumab and bevacizumab are not regarded as a cost-effective regimen in France and Thailand. In China, there are many regimens available, including sintilimab plus bevacizumab/bevacizumab biosimilar [34, 40, 41], durvalumab [34], tislelizumab [34, 42], nivolumab [34], and camrelizumab plus rivoceranib [34, 44], all of which are considered cost-effective. Nonetheless, some studies have also found that the combination of sintilimab plus bevacizumab [31, 36, 39, 45], atelizumab plus bevacizumab [31, 34, 36, 37, 39, 45], and cabozantinib plus atezolizumab [34] are not cost-effective in China.

WTP is one of the important indicators for measuring pharmacoeconomic results. It represents the amount of money the health care system or society is willing to pay to improve a person’s quality of life [50]. The level of WTP directly affects the results of cost-benefit analysis. In Zhou et al.‘s research [41], when the WTP threshold is one time the GDP per capita in China, sintilimab plus bevacizumab is not considered cost-effective. Conversely, when the WTP threshold is three times the GDP per capita, sintilimab plus bevacizumab is considered cost-effective. Therefore, the impact of WTP on pharmacoeconomic results is significant, and it can influence decision-makers’ choices of different treatment options. When developing policy and guiding practice, consideration of changes in WTP is critical to determining optimal medical decisions.

Drug prices are a major obstacle to the implementation of advanced HCC immunotherapy. In our review, atezolizumab prices ranged from $3,885.42 to $9,419.16 per 1,200 mg in cost-effectiveness studies [32, 36]. In a study conducted from a US payer perspective using atezolizumab prices of approximately $9,419.16 per 1,200 mg, atezolizumab treatment was not cost-effective [38]. In contrast, for sintilimab, most studies used prices of approximately $166.57 to $439.41 per 100 mg for analysis, but one study used a price of $656 per 100 mg [34]. This was one of the major factors causing unfavorable cost-effectiveness results.

Cost-effectiveness analyses of any type of ICI (such as sintilimab plus bevacizumab/bevacizumab biosimilar, atezolizumab plus bevacizumab, nivolumab, or tislelizumab) have the same key drivers of cost-effectiveness results, namely, the utility for PFS or OS, price, burden of disease, and fund resources. Most of the utility values are derived from the phase III clinical trials [14, 51], other literature data [52, 53], or the National Institute for Health and Care Excellence technology appraisal guidance [54, 55], since no country-specific utility data are available for countries except for France. Therefore, we do not deny that using a country-specific utility value may result in a deviation in the results [56, 57].

10 of 17 studies [29, 31–35, 38, 39, 41, 45] incorporate HRs for PFS and OS into models. Some studies use indirect comparisons because there are no direct randomized controlled trials between/among the drug groups. Most studies used a common control drug as a bridge and used the constant HR hypothesis [17, 58–60]. A subgroup analysis conducted by varying the HRs for PFS found that atezolizumab plus bevacizumab was associated with primarily negative incremental net health benefits, and the probability of cost-effectiveness was lower than 50% in most of the subgroups [35]. Indirect treatment comparisons may lead to a more optimistic/pessimistic survival rate in the experimental group when the HRs for the PFS and OS survival values are altered.

Furthermore, model structural uncertainty can potentially lead to substantial changes in cost-effectiveness results. The economic evaluations of tumor diseases have been shifted from Markov models to partitioned survival models, with some increasing [61, 62] while the rest decreasing [63, 64] the incremental QALY values. Specifically, Edward et al.‘s study contrasts a partitioned survival model with a Markov model in the context of advanced cancer [61]. The difference in ICUR between the Markov model and the partitioned survival model led to a reversal of the final economic conclusion. According to National Institute for Health and Clinical Excellence Technical Support Document Technical Support Document 19 mentioned that additional investigation is needed to determine the potential biases associated with partitioned survival models and Markov models, as well as to understand how these biases may vary depending on the specific context in which these approaches are applied [65]. In our review, 6 of the 17 studies used a Markov model, of which one study concluded that the ICI, tislelizumab, was cost-effective.

Some studies summarize the results of pharmacoeconomic scenario analyzes for ICIs as first-line treatment of advanced HCC. Through different scenario analyses, the researchers considered the impact of multiple factors on treatment options and assessed the cost-effectiveness of regimens. Among them, in some scenarios, strategies such as reducing drug dosage [40, 41] or price [37] and improving patient survival expectations [30] are adopted, and the treatment shows good cost-effectiveness. However, there are scenarios where treatment is less cost-effective when factors such as drug donation programs [36] and active treatment [39] are taken into account. In addition, studies have considered changes in treatment effectiveness under pessimistic and optimistic survival scenarios [29], as well as cost-effectiveness for different patient groups. Taken together, these studies provide an important reference for the development of first-line treatment options for advanced HCC, but multiple factors need to be considered comprehensively to formulate the best treatment strategy.

The results of this review are limited by our search strategy, inclusion criteria, the databases searched, and the time period of the search. Our study focused on literature published in the English language only. Even in the published literature, the effect sizes of the economic impact of the ICI programs may vary depending on the methodological quality of the study. Furthermore, our investigation encompassed economic assessments from varied national perspectives, including those of the US, China, Thailand, and France. It is important to note that certain data within the model, such as clinical data and utility values, were not exclusively sourced from country-specific datasets. Thus, the results should be interpreted with caution. At last, different cost-effectiveness thresholds, budget impact analysis, equity, and healthcare policies in each country can all affect the outcomes of economic evaluations. Despite these limitations, we believe we have identified and synthesized the relevant articles on the cost-effectiveness of ICIs as first-line therapy in advanced HCC.

## Conclusion

In the current context, the use of ICIs in advanced HCC treatment is unlikely to be cost-effective from social, healthcare system, payer, and patient perspectives. The findings are sensitive to price, improved survival, and utility values. National decision-makers can provide superior cost-effectiveness programs for patients by setting better drug prices.

### Electronic supplementary material

Below is the link to the electronic supplementary material.

Supplementary Material 1

## Data Availability

The datasets generated during and/or analyzed during the current study are available from the first author on reasonable request.
