# Supplementary material for: Cost-effectiveness of immune checkpoint inhibitors as a first-line therapy for advanced hepatocellular carcinoma: a systematic review
Source: Health Econ Rev. 2024 Jul 5;14:48. doi: 10.1186/s13561-024-00526-2 (PMC11225220; doi:10.1186/s13561-024-00526-2)
Supplement: Supplementary file 1 — Supplementary Material 1 [file 13561_2024_526_MOESM1_ESM.docx]

Table 1. PRISMA NMA checklist.

| **Section/Topic** | **Item #** | **Checklist item** | **Reported on page #** |
| --- | --- | --- | --- |
| **TITLE** |  |  |  |
| Title | 1 | Identify the report as a systematic review *incorporating a network meta-analysis (or related form of meta-analysis).* | Page 1 |
|  |  |  |  |
| **ABSTRACT** |  |  |  |
| Structured summary | 2 | Provide a structured summary including, as applicable:  **Background:** main objectives  **Methods:** data sources; study eligibility criteria, participants, and interventions; study appraisal; and *synthesis methods, such as network meta-analysis.*  **Results:** number of studies and participants identified; summary estimates with corresponding confidence/credible intervals; *treatment rankings may also be discussed. Authors may choose to summarize pairwise comparisons against a chosen treatment included in their analyses for brevity.*  **Discussion/Conclusions:** limitations; conclusions and implications of findings.  **Other:** primary source of funding; systematic review registration number with registry name. | Page 1-2 |
|  |  |  |  |
| **INTRODUCTION** |  |  |  |
| Rationale | 3 | Describe the rationale for the review in the context of what is already known*, including mention of why a network meta-analysis has been conducted.* | Page 3-5 |
| Objectives | 4 | Provide an explicit statement of questions being addressed, with reference to participants, interventions, comparisons, outcomes, and study design (PICOS). | Page 6 |
|  |  |  |  |
| **METHODS** |  |  |  |
| Protocol and registration | 5 | Indicate whether a review protocol exists and if and where it can be accessed (e.g., Web address); and, if available, provide registration information, including registration number. | Page 5 |
| Eligibility criteria | 6 | Specify study characteristics (e.g., PICOS, length of follow-up) and report characteristics (e.g., years considered, language, publication status) used as criteria for eligibility, giving rationale. *Clearly describe eligible treatments included in the treatment network, and note whether any have been clustered or merged into the same node (with justification).* | Page 6 |
| Information sources | 7 | Describe all information sources (e.g., databases with dates of coverage, contact with study authors to identify additional studies) in the search and date last searched. | Page 5-6 |
| Search | 8 | Present full electronic search strategy for at least one database, including any limits used, such that it could be repeated. | Page 5-6 and Supplementary Table 2 |
| Study selection | 9 | State the process for selecting studies (i.e., screening, eligibility, included in systematic review, and, if applicable, included in the meta-analysis). | Page 6 |
| Data collection process | 10 | Describe method of data extraction from reports (e.g., piloted forms, independently, in duplicate) and any processes for obtaining and confirming data from investigators. | Page 7 |
| Data items | 11 | List and define all variables for which data were sought (e.g., PICOS, funding sources) and any assumptions and simplifications made. | Page 7 |
| **Geometry of the network** | **S1** | Describe methods used to explore the geometry of the treatment network under study and potential biases related to it. This should include how the evidence base has been graphically summarized for presentation, and what characteristics were compiled and used to describe the evidence base to readers. | N/A |
| Risk of bias within individual studies | 12 | Describe methods used for assessing risk of bias of individual studies (including specification of whether this was done at the study or outcome level), and how this information is to be used in any data synthesis. | eTable 5 |
| Summary measures | 13 | State the principal summary measures (e.g., risk ratio, difference in means). *Also describe the use of additional summary measures assessed, such as treatment rankings and surface under the cumulative ranking curve (SUCRA) values, as well as modified approaches used to present summary findings from meta-analyses.* | Page 5 |
| Planned methods of analysis | 14 | Describe the methods of handling data and combining results of studies for each network meta-analysis. This should include, but not be limited to:   - *Handling of multi-arm trials;* - *Selection of variance structure;* - *Selection of prior distributions in Bayesian analyses; and* - *Assessment of model fit.* | N/A |
| **Assessment of Inconsistency** | **S2** | Describe the statistical methods used to evaluate the agreement of direct and indirect evidence in the treatment network(s) studied. Describe efforts taken to address its presence when found. | N/A |
| Risk of bias across studies | 15 | Specify any assessment of risk of bias that may affect the cumulative evidence (e.g., publication bias, selective reporting within studies). | Page 7 |
| Additional analyses | 16 | Describe methods of additional analyses if done, indicating which were pre-specified. This may include, but not be limited to, the following:   - Sensitivity or subgroup analyses; - Meta-regression analyses; - *Alternative formulations of the treatment network; and* - *Use of alternative prior distributions for Bayesian analyses (if applicable).* | N/A |
| **RESULTS†** |  |  |  |
| Study selection | 17 | Give numbers of studies screened, assessed for eligibility, and included in the review, with reasons for exclusions at each stage, ideally with a flow diagram. | Page 5-6 |
| **Presentation of network structure** | **S3** | Provide a network graph of the included studies to enable visualization of the geometry of the treatment network. | N/A |
| **Summary of network geometry** | **S4** | Provide a brief overview of characteristics of the treatment network. This may include commentary on the abundance of trials and randomized patients for the different interventions and pairwise comparisons in the network, gaps of evidence in the treatment network, and potential biases reflected by the network structure. | N/A |
| Study characteristics | 18 | For each study, present characteristics for which data were extracted (e.g., study size, PICOS, follow-up period) and provide the citations. | Page 9-12 |
| Risk of bias within studies | 19 | Present data on risk of bias of each study and, if available, any outcome level assessment. | Page 8-9 |
| Results of individual studies | 20 | For all outcomes considered (benefits or harms), present, for each study: 1) simple summary data for each intervention group, and 2) effect estimates and confidence intervals. *Modified approaches may be needed to deal with information from larger networks.* | Supplementary Table 5 |
| Synthesis of results | 21 | Present results of each meta-analysis done, including confidence/credible intervals. *In larger networks, authors may focus on comparisons versus a particular comparator (e.g. placebo or standard care), with full findings presented in an appendix. League tables and forest plots may be considered to summarize pairwise comparisons.* If additional summary measures were explored (such as treatment rankings), these should also be presented. | N/A |
| **Exploration for inconsistency** | **S5** | Describe results from investigations of inconsistency. This may include such information as measures of model fit to compare consistency and inconsistency models, *P* values from statistical tests, or summary of inconsistency estimates from different parts of the treatment network. | N/A |
| Risk of bias across studies | 22 | Present results of any assessment of risk of bias across studies for the evidence base being studied. | Page 13 |
| Results of additional analyses | 23 | Give results of additional analyses, if done (e.g., sensitivity or subgroup analyses, meta-regression analyses*, alternative network geometries studied, alternative choice of prior distributions for Bayesian analyses,* and so forth). | N/A |
|  |  |  |  |
| **DISCUSSION** |  |  |  |
| Summary of evidence | 24 | Summarize the main findings, including the strength of evidence for each main outcome; consider their relevance to key groups (e.g., healthcare providers, users, and policy-makers). | Page 17-21 |
| Limitations | 25 | Discuss limitations at study and outcome level (e.g., risk of bias), and at review level (e.g., incomplete retrieval of identified research, reporting bias). *Comment on the validity of the assumptions, such as transitivity and consistency. Comment on any concerns regarding network geometry (e.g., avoidance of certain comparisons).* | Page 21-22 |
| Conclusions | 26 | Provide a general interpretation of the results in the context of other evidence, and implications for future research. | Page 22 |
|  |  |  |  |
| **FUNDING** |  |  |  |
| Funding | 27 | Describe sources of funding for the systematic review and other support (e.g., supply of data); role of funders for the systematic review. This should also include information regarding whether funding has been received from manufacturers of treatments in the network and/or whether some of the authors are content experts with professional conflicts of interest that could affect use of treatments in the network. | Page 41 |

PICOS = population, intervention, comparators, outcomes, study design.

* Text in italics indicateS wording specific to reporting of network meta-analyses that has been added to guidance from the PRISMA statement.

† Authors may wish to plan for use of appendices to present all relevant information in full detail for items in this section.

Table 2. Search strategy

| **PubMed** |  |
| --- | --- |
| (1) | ((((((economic evaluation[Title/Abstract]) OR (cost-effectiveness[Title/Abstract])) OR (cost-benefit[Title/Abstract])) OR (cost-utility[Title/Abstract]))) AND (((advanced[Title/Abstract]) OR (unresectable[Title/Abstract])))) AND ("Carcinoma, Hepatocellular/drug therapy"[Mesh]) |
| (2) | ("2010/01/01"[Date - Publication]: "2024/04/01"[Date - Publication]) |
| (3) | (1) AND (2) |
| **Web of science** |  |
| (1) | ALL=(Carcinoma, Hepatocellular) |
| (2) | (TS=(advanced)) OR TS=(unresectable) |
| (3) | ((TS=(economic evaluation))OR TS=(cost-effectiveness)OR TS=(cost-benefit)OR TS=(cost-utility) |
| (4) | 1. AND (2) AND (3) AND (publication date: 2010-01-01 to 2024-04-01) |
| **Scopus** |  |
| (1) | ( TITLE-ABS-KEY ( drug OR therap* ) ) AND ( ( TITLE-ABS-KEY ( Carcinoma AND Hepatocellular ) ) AND ( ( TITLE-ABS-KEY ( advanced ) OR TITLE-ABS-KEY ( unresectable ) ) ) AND ( ( TITLE-ABS-KEY ( economic AND evaluation ) OR TITLE-ABS-KEY ( cost-effectiveness ) OR TITLE-ABS-KEY ( cost-benefit ) OR TITLE-ABS-KEY ( cost-utility ) ) ) ) |
| (2) | (publication date: 2010-01-01 to 2024-04-01) |
| (3) | 1. AND (2) |
| **Embase** |  |
| (1) | (‘drug OR therap*’):ti,ab,kw |
| (2) | ('unresectable hepatocellular carcinoma' OR 'advanced hepatocellular carcinoma'):ti,ab,kw |
| (3) | ('economic evaluation' OR 'cost-effectiveness' OR ‘cost-benefit’ OR ‘cost-utility’):ti,ab,kw |
| (4) | [1-1-2010]/sd NOT [1-4-2024]/sd |
| (5) | 1. AND (2) AND (3) AND (4) |
| **Cochrane** |  |
| (1) | (‘drug OR therap*’):ti,ab,kw |
| (2) | ('unresectable hepatocellular carcinoma' OR 'advanced hepatocellular carcinoma'):ti,ab,kw |
| (3) | ('economic evaluation' OR 'cost-effectiveness' OR ‘cost-benefit’ OR ‘cost-utility’):ti,ab,kw |
| (4) | (1) AND (2) AND (3) (custom range: 2010-01-01 to 2024-04-01) |

Table 3. Eligibility criteria for selecting the included studies

|  | **Inclusion criteria** | **Exclusion criteria** |
| --- | --- | --- |
| **Population** | Patients with advanced or unresectable HCC | Patients with advanced or unresectable HCC previously receiving systemic treatment |
| **Intervention** | ICIs approved by FDA for the treatment of advanced HCC | Those drugs that have been withdrawn by the FDA by the inception of the current study  TKIs approved by FDA for the treatment of advanced HCC |
| **Comparator** | All the possible comparators in the relevant studies are considered, which may include other drug interventions, non-drug interventions, placebo, and no interventions | NA |
| **Outcomes** | No restrictions are set on study outcomes. The potential relevant outcomes cover both measures of health outcomes (e.g. progression-free survival, overall survival, QALY, DALY, etc.) and economic outcomes (e.g. ICER, ICUR, etc.). | NA |
| **Study types** | Full economic evaluations in which both the costs and outcomes are evaluated and compared with alternative interventions; | Partial economic evaluations that only report outcomes unrelated to costs, health outcomes, and/or economic evaluation outcomes  Editorials, commentaries, reviews, theoretical papers, replies, viewpoints correspondences, and protocols |

NA, not applicable; ICIs, immune checkpoint inhibitors; TKIs, tyrosine kinase inhibitors; FDA: Food and Drug Administration; QALYs, quality-adjusted life-years; DALY, disability-adjusted life year; ICER, incremental cost-effectiveness ratio; ICUR, incremental cost-utility ratio;

Table 4. Results of quality assessment using the Consolidated Health Economic Evaluation Reporting Standards

|  | Item | Guidance for reporting | Percentage of studies reporting  (out of 13 full-text articles) |
| --- | --- | --- | --- |
| TITLE |  |  |  |
| Title | 1 | Identify the study as an economic evaluation and specify the interventions being compared | 100% |
| ABSTRACT |  |  |  |
| Abstract | 2 | Provide a structured summary that highlights context, key methods, results, and alternative analyses | 100% |
| INTRODUCTION |  |  |  |
| Background and objectives | 3 | Give the context for the study, the study question, and its practical relevance for decision making in policy or practice | 100% |
| METHODS |  |  |  |
| Health economic analysis plan | 4 | Indicate whether a health economic analysis plan was developed and where available | 41% |
| Study population | 5 | Describe characteristics of the study population (such as age range, demographics, socioeconomic, or clinical characteristics) | 100% |
| Setting and location | 6 | Provide relevant contextual information that may influence findings | 100% |
| Comparators | 7 | Describe the interventions or strategies being compared and why chosen. | 100% |
| Perspective | 8 | State the perspective(s) adopted by the study and why chosen. | 100% |
| Time horizon | 9 | State the time horizon for the study and why appropriate | 82% |
| Discount rate | 10 | Report the discount rate(s) and reason chosen | 100% |
| Selection of outcomes | 11 | Describe what outcomes were used as the measure(s) of benefit(s) and harm(s) | 100% |
| Measurement of outcomes | 12 | Describe how outcomes used to capture benefit(s) and harm(s) | 100% |
| Valuation of outcomes | 13 | Describe the population and methods used to measure and value outcomes | 100% |
| Measurement and valuation of resources and costs | 14 | Describe how costs were valued | 100% |
| Currency, price date, and conversion | 15 | Report the dates of the estimated resource quantities and unit costs, plus the currency and year of conversion. | 100% |
| Rationale and description of model | 16 | If modelling is used, describe in detail and why used. Report if the model is publicly available and where it can be accessed. | 100% |
| Analytics and assumptions | 17 | Describe any methods for analysing or statistically transforming data, any extrapolation methods, and approaches for validating any model used | 82% |
| Characterizing heterogeneity | 18 | Describe any methods used for estimating how the results of the study vary for sub-groups | 52% |
| Characterizing distributional effects | 19 | Describe how impacts are distributed across different individuals or adjustments made to reflect priority populations | 6% |
| Characterizing uncertainty | 20 | Describe methods to characterize any sources of uncertainty in the analysis | 100% |
| Approach to engagement with patients and others affected by the study | 21 | Describe any approaches to engage patients or service recipients, the general public, communities, or stakeholders (e.g., clinicians or payers) in the design of the study | 29% |
| RESULTS |  |  |  |
| Study parameters | 22 | Report all analytic inputs (e.g., values, ranges, references) including uncertainty or distributional assumptions. | 100% |
| Summary of main results | 23 | Report the mean values for the main categories of costs and outcomes of interest and summarise them in the most appropriate overall measure. | 100% |
| Effect of uncertainty | 24 | Describe how uncertainty about analytic judgments, inputs, or projections affect findings. Report the effect of choice of discount rate and time horizon, if applicable. | 100% |
| Effect of engagement with patients and others affected by the study | 25 | Report on any difference patient/service recipient, general public, community, or stakeholder involvement made to the approach or findings of the study | 35% |
| DISCUSSION |  |  |  |
| Study findings, limitations, generalizability, and current knowledge | 26 | Report key findings, limitations, ethical or equity considerations not captured, and how these could impact patients, policy, or practice. | 100% |
| OTHER RELEVANT INFORMATION |  |  |  |
| Source of funding | 27 | Describe how the study was funded and any role of the funder in the identification, design, conduct, and reporting of the analysis | 100% |
| Conflicts of interest | 28 | Report authors conflicts of interest according to journal or International Committee of Medical Journal Editors requirements | 100% |

Table 5. Quality assessment results of the included studies in full

|  | **Item** | **Guidance for reporting** | **Zhao, M, 2022 (China)** | **Zhou, T, 2022 (China)** | **Su, D, 2021 (US)** | **Zhang, X, 2021 (China)** | **Gaugain, L., 2023 (France)** | **Chiang, C. L, 2021 (China, Hongkong)** | **Sun, K. X, 2022 (China)** | **Li, L, 2022 (China)** | **Li, Y, 2022 (China)** | **Li, Y, 2022 (China)** | **Wen, F, 2021 (China)** | **Zhou, T, 2022 (China)** | **Liu, K. 2023 (China)** | **Zheng, Z, 2024 (China)** | **Sriphoosanaphan, 2024, (Thailand)** | **Lang W, 2024, (China)** | **Gong H, 2023, (China)** |
| --- | --- | --- | --- | --- | --- | --- | --- | --- | --- | --- | --- | --- | --- | --- | --- | --- | --- | --- | --- |
| **TITLE** |  |  |  |  |  |  |  |  |  |  |  |  |  |  |  |  |  |  |  |
| Title | 1 | Identify the study as an economic evaluation and specify the interventions being compared | Y | Y | Y | Y | Y | Y | Y | Y | Y | Y | Y | Y | Y | Y | Y | Y | Y |
| **ABSTRACT** |  |  |  |  |  |  |  |  |  |  |  |  |  |  |  |  |  |  |  |
| Abstract | 2 | Provide a structured summary that highlights context, key methods, results and alternative analyses | Y | Y | Y | Y | Y | Y | Y | Y | Y | Y | Y | Y | Y | Y | Y | Y | Y |
| **INTRODUCTION** |  |  |  |  |  |  |  |  |  |  |  |  |  |  |  |  |  |  |  |
| Background and objectives | 3 | Give the context for the study, the study question and its practical relevance for decision making in policy or practice | Y | Y | Y | Y | Y | Y | Y | Y | Y | Y | Y | Y | Y | Y | Y | Y | Y |
| **METHODS** |  |  |  |  |  |  |  |  |  |  |  |  |  |  |  |  |  |  |  |
| Health economic analysis plan | 4 | Indicate whether a health economic analysis plan was developed and where available | Y | N | N | Y | N | Y | Y | N | N | N | Y | N | N | N | Y | Y | N |
| Study population | 5 | Describe characteristics of the study population (such as age range, demographics, socioeconomic, or clinical characteristics) | Y | Y | Y | Y | Y | Y | Y | Y | Y | Y | Y | Y | Y | Y | Y | Y | Y |
| Setting and location | 6 | Provide relevant contextual information that may influence findings | Y | Y | Y | Y | Y | Y | Y | Y | Y | Y | Y | Y | Y | Y | Y | Y | Y |
| Comparators | 7 | Describe the interventions or strategies being compared and why chosen. | Y | Y | Y | Y | Y | Y | Y | Y | Y | Y | Y | Y | Y | Y | Y | Y | Y |
| Perspective | 8 | State the perspective(s) adopted by the study and why chosen. | Y | Y | Y | Y | Y | Y | Y | Y | Y | Y | Y | Y | Y | Y | Y | Y | Y |
| Time horizon | 9 | State the time horizon for the study and why appropriate | Y | N | Y | Y | Y | Y | Y | Y | Y | Y | Y | N | Y | Y | Y | Y | N |
| Discount rate | 10 | Report the discount rate(s) and reason chosen | Y | Y | Y | Y | Y | Y | Y | Y | Y | Y | Y | Y | Y | Y | Y | Y | Y |
| Selection of outcomes | 11 | Describe what outcomes were used as the measure(s) of benefit(s) and harm(s) | Y | Y | Y | Y | Y | Y | Y | Y | Y | Y | Y | Y | Y | Y | Y | Y | Y |
| Measurement of outcomes | 12 | Describe how outcomes used to capture benefit(s) and harm(s) | Y | Y | Y | Y | Y | Y | Y | Y | Y | Y | Y | Y | Y | Y | Y | Y | Y |
| Valuation of outcomes | 13 | Describe the population and methods used to measure and value outcomes | Y | Y | Y | Y | Y | Y | Y | Y | Y | Y | Y | Y | Y | Y | Y | Y | Y |
| Measurement and valuation of resources and costs | 14 | Describe how costs were valued | Y | Y | Y | Y | Y | Y | Y | Y | Y | Y | Y | Y | Y | Y | Y | Y | Y |
| Currency, price date, and conversion | 15 | Report the dates of the estimated resource quantities and unit costs, plus the currency and year of conversion. | Y | Y | Y | Y | Y | Y | Y | Y | Y | Y | Y | Y | Y | Y | Y | Y | Y |
| Rationale and description of model | 16 | If modelling is used, describe in detail and why used. Report if the model is publicly available and where it can be accessed. | Y | Y | Y | Y | Y | Y | Y | Y | Y | Y | Y | Y | Y | Y | Y | Y | Y |
| Analytics and assumptions | 17 | Describe any methods for analysing or statistically transforming data, any extrapolation methods, and approaches for validating any model used | Y | N | Y | Y | N | Y | Y | N | Y | Y | Y | Y | Y | Y | Y | Y | Y |
| Characterizing heterogeneity | 18 | Describe any methods used for estimating how the results of the study vary for sub-groups | Y | Y | Y | N | Y | Y | N | Y | Y | Y | N | N | N | N | N | Y | N |
| Characterizing distributional effects | 19 | Describe how impacts are distributed across different individuals or adjustments made to reflect priority populations | N | N | N | N | N | Y | N | N | N | N | N | N | N | N | N | N | N |
| Characterizing uncertainty | 20 | Describe methods to characterize any sources of uncertainty in the analysis | Y | Y | Y | Y | Y | Y | Y | Y | Y | Y | Y | Y | Y | Y | Y | Y | Y |
| Approach to engagement with patients and others affected by the study | 21 | Describe any approaches to engage patients or service recipients, the general public, communities, or stakeholders (e.g., clinicians or payers) in the design of the study | Y | Y | N | N | Y | Y | N | N | N | N | N | Y | N | N | Y | N | N |
| **RESULTS** |  |  |  |  |  |  |  |  |  |  |  |  |  |  |  |  |  |  |  |
| Study parameters | 22 | Report all analytic inputs (e.g., values, ranges, references) including uncertainty or distributional assumptions. | Y | Y | Y | Y | Y | Y | Y | Y | Y | Y | Y | Y | Y | Y | Y | Y | Y |
| Summary of main results | 23 | Report the mean values for the main categories of costs and outcomes of interest and summarise them in the most appropriate overall measure. | Y | Y | Y | Y | Y | Y | Y | Y | Y | Y | Y | Y | Y | Y | Y | Y | Y |
| Effect of uncertainty | 24 | Describe how uncertainty about analytic judgments, inputs, or projections  affect findings. Report the effect of choice of discount rate and time horizon,  if applicable. | Y | Y | Y | Y | Y | Y | Y | Y | Y | Y | Y | Y | Y | Y | Y | Y | Y |
| Effect of engagement with patients and others affected by the study | 25 | Report on any difference patient/service recipient, general public, community, or stakeholder involvement made to the approach or findings of the study | N | Y | N | N | Y | N | N | N | N | N | N | Y | N | Y | Y | N | Y |
| **DISCUSSION** |  |  |  |  |  |  |  |  |  |  |  |  |  |  |  |  |  |  |  |
| Study findings, limitations, generalizability, and current knowledge | 26 | Report key findings, limitations, ethical or equity considerations not captured, and how these could impact patients, policy, or practice. | Y | Y | Y | Y | Y | Y | Y | Y | Y | Y | Y | Y | Y | Y | Y | Y | Y |
| **OTHER RELEVANT INFORMATION** |  |  |  |  |  |  |  |  |  |  |  |  |  |  |  |  |  |  |  |
| Source of funding | 27 | Describe how the study was funded and any role of the funder in the identification, design, conduct, and reporting of the analysis | Y | Y | Y | Y | Y | Y | Y | Y | Y | Y | Y | Y | Y | Y | Y | Y | Y |
| Conflicts of interest | 28 | Report authors conflicts of interest according to journal or International Committee of Medical Journal Editors requirements | Y | Y | Y | Y | Y | Y | Y | Y | Y | Y | Y | Y | Y | Y | Y | Y | Y |

Table 6. Characteristics of the phase III clinical trials

| Trial name | Intervention | Sample size | Mean age (SD) | Comparators | Sample size | Mean age (SD) | Median OS (months) | HR for OS (95% CI) | Median PFS  (months) | HR for PFS (95% CI) | Median follow-up (IQR) |
| --- | --- | --- | --- | --- | --- | --- | --- | --- | --- | --- | --- |
| IMbrave150 | Atezolizumab plus Bevacizumab | 336 | 62.9 (11.9) | Sorafenib | 165 | 64.4 (10.9) | 19.2 vs 13.4 | 0.66 (0.52 to 0.85) | 6.9 vs 4.3 | 0.65 (0.53 to 0.81) | 15.6 (NR) |
| ORIENT-32 | Sintilimab plus IBI305 | 381 | 53 (NR) | Sorafenib | 191 | 54 (NR) | NR vs 10.4 | 0.57 (0.43 to 0.75) | 4.6 vs 2.8 | 0.56 (0.46 to 0.70) | 10.0 (8.5 to 11.7) |
| CheckMate 459 | Nivolumab | 371 | 63.9 (10.61) | Sorafenib | 372 | 64.5 (10.91) | 16.4 vs 14.7 | 0.85 (0.72 to 1.02) | 3.7 vs 3.8 | 0.93 (0.79 to 1.10) | 15.2 (5.7 to 28.0) |
| RATIONALE-301 | Tislelizumab | 342 | 62 (NR) | Sorafenib | 332 | 60 (NR) | 15.9 vs 14.1 | 0.85 (0.71 to 1.02) | 2.1 vs 3.4 | 1.11 (0.92 to 1.33) | NR |
| SHR-1210-III-310 | Camrelizumab plus Rivoceranib | 272 | NR | Sorafenib | 271 | NR | 22.1 vs 15.2 | 0.62 (0.49 to 0.80) | 5.6 vs 3.7 | 0.52 (0.41 to 0.65) | 14.5 (9.1 to 18.7) |
| LEAP-002 | Pembrolizumab plus Lenvatinib | 395 | 64.2 (10.9) | Lenvatinib | 399 | 64.1 (12.1) | 21.2 vs 19.0 | 0.84 (0.71 to 0.99) | 8.2 vs 8.1 | 0.83 (0.71 to 0.98) | 32.1 (29.4 to 35.3) |
| COSMIC-312 | Cabozantinib plus Atezolizumab | 432 | 65 (NR) | Sorafenib | 217 | 64 (NR) | 15.4 vs 15.5 | 0.90 (0.69 to 1.18) | 3.7 vs 3.6 | 0.63 (0.4 to 0.91) | 15·8 (14·5–17·2) |
| CARES-310 | Camrelizumab plus Rivoceranib | 272 | 58 (48–66) | Sorafenib | 271 | 56 (47–64) | 22.1 vs 15.2 | 0·62 (95% CI 0·49-0·80) | 5.6 vs 3.7 | 0·52 (95% CI 0·41-0·65) | 14·5 (IQR 9·1-18·7) |

NR, not reported; SD, standard deviation; OS: overall survival; CI: confidence interval; IQR: interquartile range

Table 7. Probabilistic sensitivity analysis parameters and summaries of economic evaluations of first-line treatment of advanced hepatocellular carcinoma

| **First author, year (country)** | **Model** | **Probabilistic sensitivity analysis parameters** | **Summary** |
| --- | --- | --- | --- |
| Zhao, M, 2022 (China) | Partitioned survival model | The results showed that, under the WTP thresholds of $33,521/QALY, the probabilities that lenvatinib, donafenib, sintilimab plus bevacizumab, and atezolizumab plus bevacizumab had economic advantages over sorafenib were 31.91%, 69.21%, 3.44%, and 0.00%, respectively. | Donafenib is still the most economical option for patients in China due to its low price. |
| Zhou, T, 2022 (China) | Partitioned survival model | The results showed that the probability of sintilimab plus bevacizumab being more cost-effective than lenvatinib was 11.6%, 55.8%, and 88.6% under the WTP thresholds of $12,516/QALY (GDP per capita in 2021), $25,031/QALY (two times the GDP per capita in 2021) and $37,547/QALY (three times the GDP per capita in 2021), respectively. | The sintilimab plus bevacizumab had a higher probability being cost-effective than lenvatinib when WTP is over $23,650/QALY in China. |
| Su, D, 2021 (US) | Partitioned survival model | The results showed that the probability of atezolizumab plus bevacizumab was cost-effective than sorafenib from 35% to 68% when the threshold value ranged from $150,000/QALY to $200,000/QALY. | Sorafenib is still the most economical option for patients in the United States due to its low price. |
| Zhang, X, 2021 (China) | Partitioned survival model | The results showed that the probability of atezolizumab plus bevacizumab being cost-effective than sorafenib was 0.6% and 5.1% under the WTP thresholds of $100,000/QALY and $150,000/QALY. | Sorafenib is still the most economical option for patients in the United States due to its low price. |
| Gaugain, L., 2023 (France) | Partitioned survival model | The results showed that the probability of atezolizumab plus bevacizumab being cost-effective than sorafenib was 24% under the WTP thresholds of $133,775/QALY. | Adjusting the survival curves with French external evidence, atezolizumab plus bevacizumab is cost-effective in France and offers better chances of survival to patients. |
| Chiang, C. L, 2021 (China, Hongkong) | Markov model | NR | Sorafenib is still the most economical option for patients in the United States due to its low price. |
| Sun, K. X, 2022 (China) | Markov model | In China: The results showed that, under the WTP thresholds of $11,101.70/QALY, the probabilities that lenvatinib, and sintilimab plus bevacizumab, had economic advantages over sorafenib were 95%, and 0.1%, respectively.  In the United States: The results showed that the probability of lenvatinib being cost-effective than sorafenib was 100% under the WTP thresholds of $69375.0/QALY. | Lenvatinib is the most economical option for Chinese and US payers. |
| Li, L, 2022 (China) | Partitioned survival model | The results showed that, under the WTP thresholds of $33,500/QALY, the probabilities that atezolizumab plus bevacizumab, and sintilimab plus bevacizumab, had economic advantages over sorafenib were 0.4%, and 15.4%, respectively. | The sintilimab plus bevacizumab had a higher probability being cost-effective than sorafenib in China, if sintilimab PAP is considered. |
| Li, Y, 2022 (China) | Partitioned survival model | The results showed that the probability of nivolumab being cost-effective than sorafenib was 10.38% under the WTP thresholds of $150,000/QALY. | Sorafenib is still the most economical option for patients in the United States due to its low price. |
| Li, Y, 2022 (China) | Partitioned survival model | The results showed that the probability of atezolizumab plus bevacizumab being cost-effective than nivolumab was 25% and 78% under the WTP thresholds of $100,000/QALY and $150,000/QALY. | The atezolizumab plus bevacizumab had a higher probability being cost-effective than nivolumab when WTP is over $150,000/QALY in the United States. |
| Wen, F, 2021 (China) | Markov model | In China: The results showed that the probability of atezolizumab plus bevacizumab being cost-effective than sorafenib was 10% under the WTP thresholds of $28,527/QALY.  In the United States: The results showed that the probability of atezolizumab plus bevacizumab being cost-effective than sorafenib was 48.2% under the WTP thresholds of $150,000/QALY. | Sorafenib is the most economical option for Chinese and US payers. |
| Zhou, T, 2022 (China) | Partitioned survival model | The results showed that the probability of sintilimab plus bevacizumab being more cost-effective than sorafenib was 30%, 61%, and 77% under the WTP thresholds of $11,197/QALY (GDP per capita), $22,395/QALY (2 times the GDP per capita), and $33,592/QALY (3 times the GDP per capita), respectively. | The sintilimab plus bevacizumab had a higher probability being cost-effective than sorafenib when WTP is over $33,592/QALY in China. |
| Liu, K. 2023 (China) | Markov model | The results showed that the probability of tislelizumab, lenvatinib, donafenib,nivolumab, camrelizumab plus rivoceranib, durvalumab, sintilimab plus a bevacizumab biosimilar, pembrolizumab plus lenvatinib, sorafenib plus erlotinib, sunitinib, atezolizumab plus bevacizumab, brivanib, linifanib, sorafenib plus doxorubicin, cabozantinib plus atezolizumab therapies being cost-effective were 96.7%, 86.3%, 84.5%, 82.4%, 75.4%, 73.6%, 60.0%, 24.0%, 0.3%, 1.6%, 0.9%, 0%, 0%, 0%, and 0% compared with sorafenib at a WTP threshold of $37,653/QALY, respectively | Results demonstrate that tislelizumab or lenvatinib versus sorafenib as first-line systematic treatment were dominant. Tislelizumab is the most cost-effective in China. |
| Zheng, Z 2024 (China) | Partitioned survival model | At a WTP threshold of $37304.346 per QALY, the probability of considering the tislelizumab regimen as a more cost-effective option is exceedingly high, at  approximately 99.99% compared to the sorafenib group. | Tislelizumab holds promise as a cost effective first-line treatment option for advanced HCC in comparison  to sorafenib. |
| Sriphoosanaphan, 2024, (Thailand) | Markov model | With the Thai ceiling threshold, the probability that atezolizumab plus bevacizumab was cost-effective for the treatment of uHCC patients was 0%. | The atezolizumab plus bevacizumab is not cost-effective in Thailand at the current price and poses budgetary challenges. |
| Lang W, 2024, (China) | Markov model | Probabilstic sensitivity analyses showed that the probabilities that camrelizumab plus rivoceranib was cost efective were 61.27%, 51.46%, and 82.78% for unresectable HCC with ALBI of any grade, grade 1, and grade 2, compared to a WTP threshold of three times GDP per capita, which was $35,864.61 in China. | Camrelizumab plus rivoceranib was more cost efective than sorafenib as frst-line therapy for unresectable HCC in the Chinese setting. |
| Gong H, 2023, (China) | Partitioned survival model | Probabilstic sensitivity analyses showed that the probabilities that Lenvatinib, sintilimab plus bevacizumab, and atezolizumab plus bevacizumab were cost-efective were 0% for unresectable HCC compared to a WTP threshold of three times GDP per capita. | Lenvatinib, sintilimab plus bevacizumab, and atezolizumab plus bevacizumab were not costefective at a willingness-to-pay threshold of US$36,600 per QALY. |

NR, Not reported; PAP, Patient Assistance Program; QALYs, quality-adjusted life-years, WTP, willing to pay; GDP, gross domestic product; HCC: hepatocellular carcinoma;
